# Modeling Spatial and Temporal Variability of Residential Air Exchange Rates for the Near-Road Exposures and Effects of Urban Air Pollutants Study (NEXUS)

**DOI:** 10.3390/ijerph111111481

**Published:** 2014-11-07

**Authors:** Michael S. Breen, Janet M. Burke, Stuart A. Batterman, Alan F. Vette, Christopher Godwin, Carry W. Croghan, Bradley D. Schultz, Thomas C. Long

**Affiliations:** 1 National Exposure Research Laboratory, United States Environmental Protection Agency, Research Triangle Park, NC 27711, USA; E-Mails: burke.janet@epa.gov (J.M.B.); croghan.carry@epa.gov (C.W.C.); schultz_brad@yahoo.com (B.D.S.); 2 Environmental Health Sciences, University of Michigan, Ann Arbor, MI 48109, USA; E-Mails: stuartb@umich.edu (S.A.B.); ccgodwin@umich.edu (C.G.); 3 Immediate Office of the Assistant Administrator, United States Environmental Protection Agency, Research Triangle Park, NC 27711, USA; E-Mail: vette.alan@epa.gov; 4 National Center for Environmental Assessment, United States Environmental Protection Agency, Research Triangle Park, NC 27711, USA; E-Mail: long.tom@epa.gov

**Keywords:** air exchange rate modeling, air pollution, health, exposure

## Abstract

Air pollution health studies often use outdoor concentrations as exposure surrogates. Failure to account for variability of residential infiltration of outdoor pollutants can induce exposure errors and lead to bias and incorrect confidence intervals in health effect estimates. The residential air exchange rate (AER), which is the rate of exchange of indoor air with outdoor air, is an important determinant for house-to-house (spatial) and temporal variations of air pollution infiltration. Our goal was to evaluate and apply mechanistic models to predict AERs for 213 homes in the Near-Road Exposures and Effects of Urban Air Pollutants Study (NEXUS), a cohort study of traffic-related air pollution exposures and respiratory effects in asthmatic children living near major roads in Detroit, Michigan. We used a previously developed model (LBL), which predicts AER from meteorology and questionnaire data on building characteristics related to air leakage, and an extended version of this model (LBLX) that includes natural ventilation from open windows. As a critical and novel aspect of our AER modeling approach, we performed a cross validation, which included both parameter estimation (*i.e.*, model calibration) and model evaluation, based on daily AER measurements from a subset of 24 study homes on five consecutive days during two seasons. The measured AER varied between 0.09 and 3.48 h^−1^ with a median of 0.64 h^−1^. For the individual model-predicted and measured AER, the median absolute difference was 29% (0.19 h^‑1^) for both the LBL and LBLX models. The LBL and LBLX models predicted 59% and 61% of the variance in the AER, respectively. Daily AER predictions for all 213 homes during the three year study (2010–2012) showed considerable house-to-house variations from building leakage differences, and temporal variations from outdoor temperature and wind speed fluctuations. Using this novel approach, NEXUS will be one of the first epidemiology studies to apply calibrated and home-specific AER models, and to include the spatial and temporal variations of AER for over 200 individual homes across multiple years into an exposure assessment in support of improving risk estimates.

## 1. Introduction

Numerous air pollution epidemiology studies have found associations between ambient concentrations and adverse health effects [1,2]. Due to various challenges with personal exposure measurements (e.g., cost, participant burden), these health studies often use outdoor air monitors as exposure surrogates, which can: (1) introduce negative bias in health effect estimates due to time spent in indoor microenvironments with ambient-source pollutant concentrations that can be substantially attenuated from outdoor levels [3,4], and (2) increase confidence intervals of health effect estimates by not accounting for building-to-building and temporal variability of this attenuation [4]. To help improve health effect estimates, we are developing an air pollution exposure model for individuals (EMI) in health studies [5,6,7,8]. The EMI predicts personal exposures based on outdoor concentrations, meteorology, questionnaire information (e.g., building characteristics, occupant behavior related to building operation), and time-location information. A critical aspect of EMI is the air exchange rate (AER) of individual homes, which is the rate of exchange of indoor air with outdoor air. In addition, AERs have been applied as a covariate or modifying factor in air pollution epidemiology studies, showing the importance of this variable [9,10].

This study addresses the cross-validation and application of residential AER models, and specifically the AER predictions for the Near-Road Exposures and Effects of Urban Air Pollutants Study (NEXUS) [5]. The goal of NEXUS is to examine traffic-related air pollution exposures and respiratory effects in asthmatic children living near major roads in Detroit, Michigan (MI).

The AER affects both the steady-state (*i.e.*, long-term average) and dynamic (*i.e.*, time-varying) behaviors of indoor air pollutant concentrations, and the resulting exposures [11]. For example, assume that outdoor concentrations, *C*_in_ss_ are under steady-state conditions (*i.e.*, short-term changes of concentrations are considered negligible compared with long-term average concentrations), then the steady-state indoor concentrations of outdoor-generated air pollutants *C*_in_ss_ can be described by:

*C*_in_ss_ = *F*_inf_ × *C*_out_ss_ (1)

where *F*_inf_ is the fraction of *C*_out_ss_ that enters and remains airborne indoors (infiltration factor) defined as:

*F*_inf_ = *P* × AER/(AER + *k*_d_) (2)

where *P* is the penetration coefficient, and *k*_d_ is the indoor loss rate. Setting *P* = 0.9 and *k*_d_ = 1.0 h^‑1^ based on reported values for particulate matter (diameter = 2.5 µm; PM_2.5_), *C*_in_ss_ for a tight (AER = 0.1 h^‑1^) and leaky (AER = 3.0 h^‑1^) building is 0.08 and 0.68 times *C*_out_ss_, respectively. Therefore, the AER can substantially affect *C*_in_ss_. Furthermore, studies examining particulate matter show that the AER can explain a substantial amount of the variability of *F*_inf_ [12,13,14]. For time-varying outdoor concentrations *C*_out_ (e.g., traffic), indoor concentrations *C*_in_ can be described by the dynamic mass balance equation:

d*C*_in_/dt = *P* × AER × *C*_out_ – (AER + *k*_d_) × *C*_in_ (3)

Measurements of *C*_out_ and *C*_in_ for time-varying traffic pollutants show that the dynamic behavior of *C*_in_ depends on the AER [15]; for example, *C*_in_ increases more slowly and reaches lower peak levels for tighter buildings [16].

For gaseous pollutants with *k*_d_ > 0 (e.g., ozone), *F*_inf_ depends on AER [17]. For gases with negligible *k*_d_ (e.g., carbon monoxide) compared with AER, *C*_in_ss_ can be considered independent of the AER based on Equation (2) (*F*_inf_ = *P*) [18]. However, for outdoor pollutants that vary with time (e.g., traffic), time-varying *C*_in_ (Equation (3)) depends on AER even when *k*_d_ is negligible compared with AER [15].

A residential AER model has several benefits for exposure assessments in health studies. First, the AER is a key determinant for the entry of outdoor-generated air pollutants and the removal of indoor-generated air pollutants [11,19]. Since people in the United States spend approximately 66% of their time indoors at home [20,21], the residential AER is a critical parameter for air pollution exposure models. Costs and participant burden often limit the number of AER measurements. Therefore, a residential AER model integrated within exposure models can be a feasible method to predict exposure metrics for epidemiological analysis. Second, an AER model can reduce the uncertainty of exposure models by accounting for factors that influence the house-to-house (spatial) and temporal variability of the AER. These factors include the physical driving forces of the airflows (e.g., indoor-outdoor temperature differences, wind speed), building characteristics (e.g., local wind sheltering, building height, tightness of the building envelope), and occupant behavior (e.g., opening windows). Spatial and temporal differences in weather, building characteristics, and occupant behavior can produce substantial AER variations. The resulting spatial and temporal variations in exposure may help explain the impact of AER for individuals with exceptionally high and low exposures. Also, predicting the AER variability can help reduce exposure misclassifications, and the resulting errors in health effect estimates.

Various AER models are described in the literature [11]. The Lawrence Berkeley Laboratory (LBL) model is widely used to predict residential AER [22]. The LBL model predicts the AER due to airflow through small unintentional openings (*i.e.*, leakage), but does not account for the airflow through large controllable openings (*i.e.*, natural ventilation), such as open windows. Previously, we addressed this limitation by extending the LBL model (LBLX) to predict natural ventilation airflow [7]. In this study, we used the previously developed LBL and LBLX models, which were linked with a leakage area model, to predict the AER from questionnaire and weather data [7]. The LBL model was used for all homes, and the LBLX model was used for a subset of homes with window opening data, as described below.

The NEXUS design includes the development of various tiers of modeled exposure metrics for traffic-related air pollutants, and the use of measurements from a subset of homes for model calibration (*i.e.*, parameter estimation) and evaluation [5]. This paper focuses on modeling the residential AER. We used NEXUS questionnaires and airport weather data as inputs for the AER models, and AER measurements from a subset of homes for parameter estimation and model evaluation. Below, we first describe the NEXUS design, and then describe the AER models, methods for parameter estimation and model evaluation, and development of daily AER predictions for the three year health study.

## 2. Methods

### 2.1. NEXUS Design

NEXUS was designed to examine the relationship between exposures to traffic-related air pollutants and respiratory outcomes in a cohort of children with asthma living near major roads in Detroit, MI, USA [5]. For this community-based participatory research study, children from 6 to 14 years of age with asthma or symptoms of asthma were recruited based on the proximity of their home to major roads according to three traffic categories: (1) high diesel/high traffic (HTHD), (2) high traffic/low diesel (HTLD), and (3) low traffic/low diesel (LTLD) [5]. A total of 147 children participated in the study from September 2010 to December 2012. Since children moved during the study, a total of 213 residences were considered, which included 203 detached homes, nine apartments, and one townhome. The study population consisted of 98 homes in the high traffic categories (52 in HTHD, 46 in HTLD) and 115 homes in the low traffic category (LTLD).

An overview of the exposure assessment method in NEXUS has been previously described [5]. Residential indoor, residential outdoor, school outdoor, and near-highway air monitoring was performed during two seasonal intensive field sampling periods: 25 September to 11 November 2010 (Fall 2010) and 28 March to 4 May 2011 (Spring 2011). The fall and spring are peak seasons for respiratory viruses that can induce asthma symptoms. A subset of 24 homes was selected for residential monitoring during the seasonal intensives based on the traffic characteristics of nearby roads, and consisted of 12 homes in the high traffic categories (seven in HTHD, five in HTLD) and 12 homes in the low traffic category (LTLD). A maximum of four residences were monitored simultaneously during a five day period.

Daily 24 h average AERs were measured for five consecutive days during the season intensives in the 24 homes using a perfluorocarbon tracer (PFT) method [23,24]. The Brookhaven National Laboratory (BNL, Upton, NY, USA) prepared the tracer sources and receptor tubes, and provided guidance on the number of tracers sources required in each home. Sources were placed in the homes 24 h before the first day of measurement to allow for sufficient distribution. The reported accuracy (based on known AER), precision (based on replicate measurements), and limits on the PFT-derived AER measurements for occupied homes are estimated to be 20%–25%, 5%–15%, and 0.2–5.0 h^−1^, respectively [19,25,26].

These AER measurements were used for parameter estimation and evaluation of the AER model, as described below. Input data for the AER models were obtained for meteorology, housing characteristics, household income, and occupant behavior. Meteorological measurements included local airport temperature and wind speed. During the seasonal intensives on days with residential measurements, indoor temperatures were measured and occupants recorded when certain activities related to housing operation were performed, including opening windows.

### 2.2. AER Model Overview

The exchange of outdoor air with air inside occupied spaces of buildings can be separated into three categories: leakage, natural ventilation, and mechanical ventilation [19]. Leakage is the airflow through unintentional openings in the building envelope (e.g., small cracks around windows, exterior doors, joints between exterior walls and floors). Natural ventilation is the intentional airflow through controlled openings in the building envelope (e.g., open windows and doors). Mechanical ventilation is the airflow induced by outdoor-vented fans. For this study, we used two AER models, one model that includes leakage (LBL) and another model that includes both leakage and natural ventilation (LBLX) [7,11]. Mechanical ventilation was not considered since detailed information on the specific type and operation of outdoor-vented fans was unavailable from NEXUS.

The driving mechanism for airflows are pressure differences across the building envelope [11,19]. The pressure differences for leakage and natural ventilation are driven by indoor-outdoor temperature differences (stack effect) and wind (wind effect). For this study, the LBL and LBLX models include the stack and wind effects based on local airport temperature and wind speed, and building characteristics (e.g., building height and wind sheltering from nearby structures) that modify the stack and wind effect-driving forces.

Mechanistic AER models, which account for the physical driving forces of the airflows (*i.e.*, stack and wind effect) can be classified as single-zone and multizone models [11]. Single-zone models predict the AER for a whole building represented as a single, well-mixed compartment. Multizone models are required for buildings that need to be represented by a series of interconnected compartments with distinct pressures and temperatures. The LBL and LBLX models are single-zone models that are appropriate for buildings with no internal resistance to airflow, such as the homes included in this study.

We developed a computer simulation for the LBL and LBLX models linked to a leakage area model. First, parameters for the leakage area model were estimated using the LBLX model and the AER measurements and window opening data from a subset of homes. Then, daily (24 h average) AER predictions were developed for every home for the three year health study. Since window opening data was not available for the three year study, we used the LBL model to develop AER predictions for the health study. Below, we first describe the AER models, and the method for parameter estimation and model evaluation. The complete method and subsequent analysis were implemented using MATLAB software (version R2014a, Mathworks, Natick, MA, USA).

### 2.3. LBL Leakage Model

The LBL and LBLX models were previously described and evaluated for homes in central North Carolina [7]. Briefly, the LBL model predicts the AER due to leakage, and assumes the building is a single, well-mixed compartment. The leakage airflow *Q*_LBL_ is calculated as:

(4) $$Q_{\text{LBL}}=A_{\text{leak}}\sqrt{k_{\text{s}}\left|T_{\text{in}}-T_{\text{out}}\right|+k_{\text{w}}U^{2}}$$

where *A*_leak_ is the effective air leakage area, *k*_s_ is the stack coefficient, *k*_w_ is the wind coefficient, *T*_in_ and *T*_out_ are the average indoor and outdoor temperatures over time interval of calculation, respectively, and *U* is the average wind speed over time interval of calculation. The stack and wind effects are the first and second terms within the square root in Equation (4), respectively. The AER is calculated as *Q*_LBL_ divided by the building volume *V*.

The AER has two parameters (*k*_s_ and *k*_w_) and five inputs (*A*_leak_, *T*_in_, *T*_out_, *U*, and *V*). Parameters *k*_s_ and *k*_w_ were set to literature-reported values based on house-specific information on house height (number of stories) and local wind sheltering (Supplementary Material Table S1–S3). The number of stories and local wind sheltering were determined from aerial and street‑level images in Google Earth (version 7.1.2.2041; Google, Mountain View, CA, USA). We used house numbers visible in street-level images to verify the study participant homes. To determine *V*, we multiplied the floor area *A*_floor_ by the measured ceiling height (typically 2.44 m, 8 ft). The *A*_floor_ were both measured and obtained from online city and real estate databases of property records (BS&A Software, Bath, MI, USA; Zillow, Seattle WA, USA; Trulia, San Francisco, CA, USA).

We determined *T*_out_ and *U* (10 m elevation) from hourly measurements at the Detroit Metro Airport in Detroit, MI, USA. For parameter estimation, we calculated the 24 h average *T*_out_ and *U* time-matched to the 24 h average AER measurements. To develop AER predictions for all homes across the three year study period, we used hourly *T*_out_ and *U* to predict hourly AER, and then calculated daily (24 h average) AER.

We determined *T*_in_ from continuous (5 min) indoor measurements. For parameter estimation, we calculated the 24 h average *T*_in_ time-matched to the 24 h average AER measurements. For developing AER predictions for all homes across the three year study period, we set *T*_in_ to the 24 ºC, which is the overall median of 1 h average *T*_in_ from a subset of 59 homes across 6 seasons. We used a constant value for *T*_in_ since the seasonal medians of the *T*_in_ did not vary substantially (24, 24, 24, 25, 23, 23 ºC in fall 2010, winter 2010, spring 2011, summer 2011, fall 2011, winter 2011; respectively).

We estimated *A*_leak_ with a literature-reported leakage area model [7,27]. The *A*_leak_ is calculated as:

*A*_leak_ = *NL*/*NF* (5)

where *NL* is the normalized leakage and *NF* is the normalization factor. Using a classification tree analysis, Chan *et al.* determined the most important factors associated with normalized leakage were year built *Y*_built_, floor area *A*_floor_, and housing type (e.g., low-income homes, conventional homes) [27]. Their analysis was based on homes built between 1895 and 2000, which is similar to NEXUS homes built between 1888 and 2007 (Table S4). Therefore, *NL* is defined as:

*NL* = exp(*β*_0_ + *β*_1_*Y*_built_ + *β*_2_*A*_floor_) (6)

where *β*_0_, *β*_1_, and *β*_2_ are the regression parameters. In addition, one set of regression parameters are estimated for low-income homes, and another set of parameters for conventional homes. The *NF* is defined as:

*NF* = (1000/*A*_floor_) × (*H*/2.5)^0.3^ (7)

where *H* is the building height. We set *H* to the number of stories multiplied by a story height of 2.5 m and adding a roof height of 0.5 m [7]. The *A*_floor_ was obtained as described above and *Y*_built_ was obtained from online city and real estate databases of property records (BS&A Software, Bath, MI, USA; Zillow, Seattle WA, USA; Trulia, San Francisco, CA, USA).

### 2.4. LBLX Leakage and Natural Ventilation Model

The LBLX model predicts the AER due to leakage and natural ventilation. The airflow is calculated as:

(8) $$Q_{\text{LBLX}}=\sqrt{Q_{\text{LBL}}^{2}+Q_{\text{nat}}^{2}}$$

where *Q*_LBL_ is the leakage airflow as defined above, and *Q*_nat_ is the natural ventilation airflow through open windows [7]. The AER is calculated as *Q*_LBLX_ divided by V.

The airflow for natural ventilation *Q*_nat_ is calculated as:

(9) $$Q_{\text{nat}}=\sqrt{Q_{\text{nat}\_\text{wind}}^{2}+Q_{\text{nat}\_\text{stack}}^{2}}$$

where *Q*_nat,wind_ and *Q*_nat,stack_ are the airflows from the wind and stack effects, respectively. The *Q*_nat_wind_ is defined as:

*Q*_nat_wind_ = *C*_v_*A*_nat_*U* (10)

where *C*_v_ is the effectiveness of the openings, and *A*_nat_ is the area of the inlet openings. Using the literature-reported method, we set *C*_v_ to 0.30 and *A*_nat_ to one-half of the total area of window openings [7]. We calculated the 24 h average total area of window openings from daily window opening data (number of windows opened multiplied by fraction of day) multiplied by window opening area of 0.06 m^2^ (derived from literature-reported window width of 0.6 m and height of 0.1 m) [7]. The Q_nat,stack_ is defined as:

(11) $$Q_{\text{nat}\_\text{stack}}=\;\frac{C_{\text{D}}A_{\text{nat}}\sqrt{2g\text{Δ}H_{\text{NPL}}\left|T_{\text{in}}-T_{\text{out}}\right|}}{max\left\{T_{\text{in}},T_{\text{out}}\right\}}$$

where *C*_D_ is the discharge coefficient for the openings, g is the gravitational acceleration, Δ*H*_NPL_ is the height from midpoint of lower window opening to the neutral pressure level (NPL) of the building, and max{*T*_in_, *T*_out_} is the maximum value between *T*_in_ and *T*_out_. Using literature-reported values, we set *C*_D_ to 0.65, the midpoint of lower window opening to 0.91 m, and the NPL to one-half of the building height [7]. The building height is set to the number of stories multiplied by a story height of 2.5 m and adding a roof height of 0.5 m.

### 2.5. Parameters for A_leak_ and Cross Validation

We estimated the parameters (*β*_0_, *β*_1_, and *β*_2_) for *A*_leak_ (Equation (6)) using the AER measurements. The subset of homes with measured AERs consisted of a cluster of 23 older homes built between 1900 and 1969 (median 1942), and one newer home built in 1997 (Supplementary Material Figure S1). Since the cluster of 23 homes were substantially older than the home built in 1997, we used the cluster of 23 homes for parameter estimation. We then applied the estimated parameters for all homes built in 1979 or before. For homes built after 1979, we used literature-reported parameters [7]. This cutoff of 1979 was based on 10 years after 1969, which is the upper range of the cluster of homes used for parameter estimation.

The literature-reported parameters (*β*_0_, *β*_1_, *β*_2_) were previously estimated for low-income homes and conventional homes [7,27]. Low-income homes are residences with household incomes below 125% of the poverty guideline. In this study, household incomes were collected for all homes.

We performed a leave-one-home-out jackknife method to estimate parameters (*β*_0_, *β*_1_, *β*_2_) and cross validation for model evaluation [28,29,30]. Since the subset of homes with AER measurements had daily window opening data, the LBLX model was used for parameter estimation, and both the LBLX and LBL models were evaluated. We estimated parameters with a subsample of data (training sample) and evaluated the models with the remaining data (validation sample). We removed all samples from one home at a time (validation sample) and estimated parameters with the remaining subsample of data (training sample). We then evaluated the models with the validation sample. This process was performed independently for the low-income homes (n = 17) and conventional homes (n = 6) to yield two sets of parameters. Each of the 23 homes was used as a validation sample to yield 17 and 6 parameter sets for low-income and conventional homes, respectively. The jackknife estimates were then determined for the low-income homes and the conventional homes (Supplementary Material). The leave-one-home-out jackknife method was used for parameter estimation to account for repeated AER measurements at the homes.

Each parameter set was estimated using the least-squares method. Let *Y*(*x*, *d; β*) be the LBLX model-predicted AER in the *x*th home on the *d*th day with parameter set *β* = (*β*_0_, *β*_1_, *β*_2_). Let *Y_x,d_* be the measured AER in the *x*th home on the *d*th day. Then, the least squares estimate, β* = (*β*_0_*, *β*_1_*, *β*_2_*) is the parameter values *β* which minimize the cost function:

(12) $$J\left(\underline{\beta }\right)={\text{∑}}_{x=1}^{N}{\text{∑}}_{d=1}^{M}{\left[\;Y\left(x,d;\beta \right)-Y_{x,d}\right]}^{2}$$

where *N* is the number homes, and *M* is the number of days with AER measurements in the *x*th home.

Parameters were estimated with an iterative optimization algorithm. We chose the Nelder-Mead simplex method for its relative insensitivity to the initial parameters values compared with other common methods, such as Newton’s method, and its robustness to discontinuities [31]. Initial parameter values were set to literature-reported parameters [7]. Convergence to the solution was confirmed after the parameter search terminated.

### 2.6. Model Evaluation Metrics

For model evaluation, we evaluated the differences between individual model-predicted AER ( Y(x,d;β*) ) and measured AER (*Y_x,d_*) using two metrics: relative difference ε (%) and absolute difference Δ (1/h). These metrics are calculated as:

(13) $$\text{ε}=100\left(\frac{Y(x,d;\underline{\beta }*)-Y_{x,d}}{Y_{x,d}}\right)$$

(14) $$\text{Δ}=Y(x,d;\underline{\beta }*)-Y_{x,d}$$

The absolute difference Δ provides the amount of deviation, and the relative difference ε indicates whether Δ is small or large relative to the measured AER. However, for measured AER with low values, a minor deviation could yield a large ε. In this case, Δ is more meaningful than ε for model evaluation. Therefore, both ε and Δ are used in this study. A positive value for ε and Δ indicates that the model overestimated the measured AER, while a negative value indicates underestimation. Since ε and Δ indicate the bias (*i.e.*, overestimation or underestimation), we also calculated the absolute values |ε| and |Δ| to quantify the magnitude of deviation.

To compare the modeled and measured AER, we also calculated Pearson and Spearman correlation coefficients. To account for the repeated AER measurements at the homes, we calculated weighted correlation coefficients [32]. First, each measurement and model prediction for a given home is replaced with the average value for that home. Then, the correlation coefficients were calculated from the revised values. To determine the amount of variation explained by the AER models, we calculated the coefficient of determination (R^2^) as defined by the square of the Pearson correlation coefficient.

We also investigated the AER by road type since the AER could vary between road types due to differences in building characteristics that impact the leakage area (e.g., year built, floor area, housing type), and the stack and wind effects (e.g., number of stories, wind sheltering). Also, we plan to apply the AER models to predict indoor home concentrations of traffic-related pollutants, and examine the concentration differences between the road types. Therefore, understanding the AER for each road type will help determine the influence of the AER on the pollutant concentrations for each road type.

## 3. Results

For the subset of 24 homes with AER measurements, summary statistics are provided for the number of homes, number of days windows opened, daily measured AER in the two seasons and three road type classifications (Table 1), and building characteristics (Supplementary Material Table S4). Across the 24 homes in the fall and spring, the measured AER varied between 0.09 h^−1^ (minimum) to 3.48 h^‑1^ (maximum) with a median of 0.64 h^‑1^. Between the fall and spring, there was no substantial difference in the median AER (0.63 h^−1^ in fall, 0.67 h^−1^ in spring). For the road types, the median AER were highest for HTHD (0.79 h^−1^) and lowest for HTLD (0.49 h^−1^).

The estimated leakage area (*A*_leak_) model parameters for older homes are shown in Table 2. The literature-reported parameters *β*_0_ (low-income and conventional), *β*_1_ (low-income) and *β*_1_ (conventional) for newer homes (Table 3) were different (at 95% confidence level) from the corresponding estimated parameters for older homes (Table 2).

### 3.1. Model Evaluation

Overall, the modeled AERs matched the AERs measured in fall 2010 and spring 2011. Summary statistics are provided for the distributions of the modeled and measured AER (Table 1, Supplementary Material Table S5 and S6). For the LBLX model, the modeled and measured AER had similar overall medians of 0.64, 0.65 h^−1^, 25th percentiles of 0.45, 0.42 h^−1^, and 75th percentiles of 0.99, 0.99 h^−1^, respectively. For the LBL model, the AER had overall median of 0.64 h^−1^, 25th and 75th percentiles of 0.43 and 0.97 h^−1^, respectively, which were slightly lower than the LBLX model.

**Figure 1:**
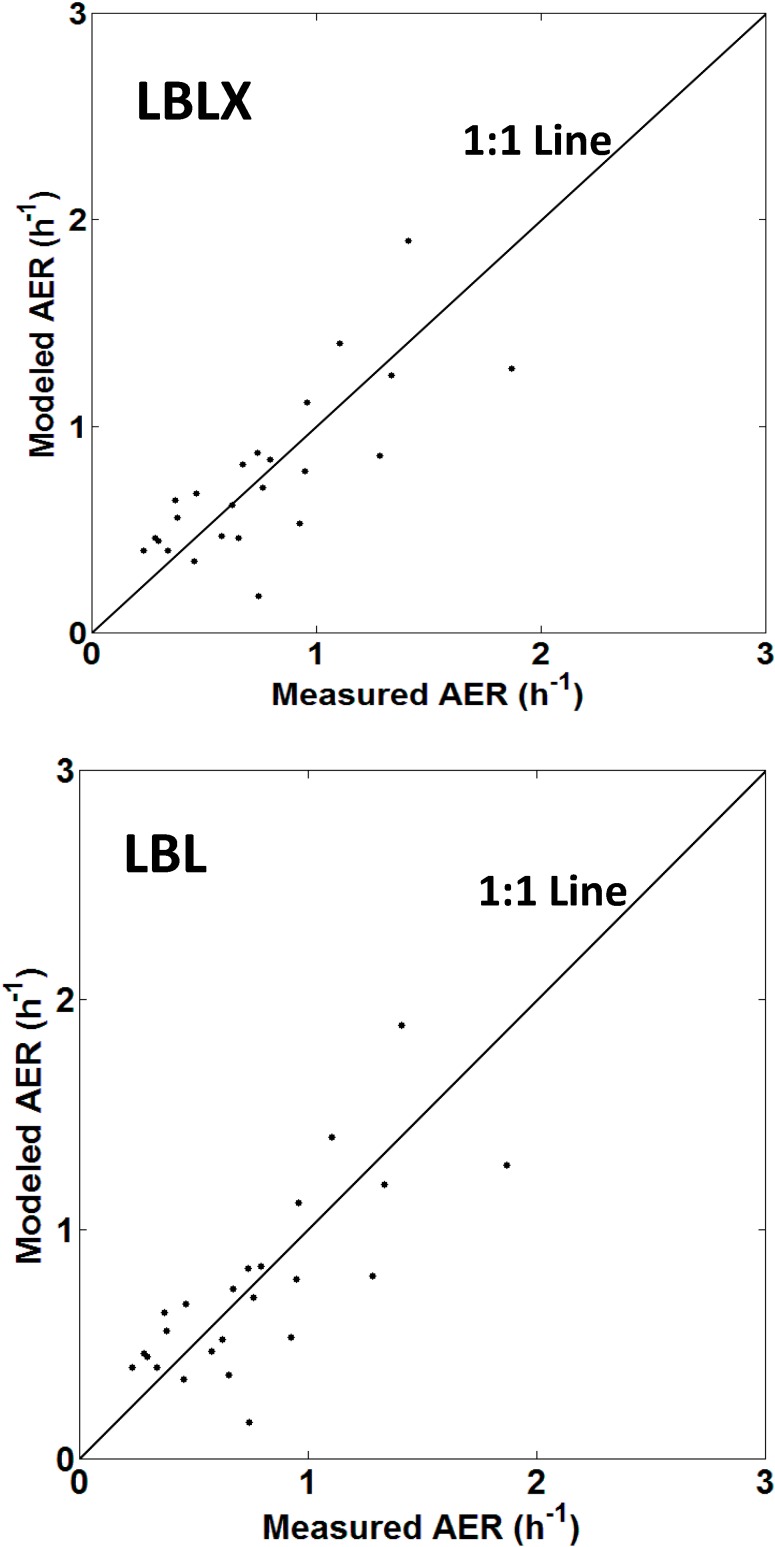
Scatter plots of the LBLX (**top**) and LBL (**bottom**) model-predicted and measured AER for each home. The points are average AER values for each home. Points above and below the 1:1 line indicate model overestimation and underestimation, respectively.

Scatter plots of the modeled and measured AER for each home are shown (Figure 1, Supplementary Material Figure S2). Overall, the weighted Pearson and Spearman correlation coefficients were 0.78 (R^2^ = 0.61) and 0.81 for the LBLX model, and 0.77 (R^2^=0.59) and 0.79 for the LBL model, respectively.

**Table 1:** Number of homes, number of days windows opened, and summary statistics for measured 24 h average air exchange rates.

Season: Year ^1^ or Road Type Classification of Home	Number of Homes	Number of Days Windows Opened ^2^	Air Exchange Rates (h^−1^)											
			Sample Size	Mean	SD	Min	p5	p10	p25	p50	p75	p90	p95	Max
Fall 2010	24	19 (16%)	119	0.74	0.56	0.09	0.12	0.17	0.41	0.63	0.97	1.21	1.69	3.48
Spring 2011	17	9 (12%)	78	0.83	0.48	0.25	0.32	0.35	0.45	0.67	1.06	1.66	1.81	2.05
HTHD **^3^**	7	12 (22%)	55	1.00	0.73	0.11	0.14	0.39	0.53	0.79	1.17	2.01	2.70	3.48
HTLD **^3^**	5	2 (5%)	44	0.65	0.41	0.09	0.13	0.16	0.35	0.49	0.96	1.18	1.52	1.82
LTLD **^3^**	12	14 (14%)	98	0.70	0.39	0.09	0.20	0.25	0.43	0.64	0.91	1.23	1.51	1.80
All	24	28 (14%)	197	0.77	0.53	0.09	0.16	0.25	0.42	0.64	0.99	1.43	1.81	3.48

Notes: ^**1**^ Fall: September, October, and November; spring: March, April, and May; **^2^** Percentage of days windows opened relative to corresponding sample size are shown in parentheses; **^3^** HTHD: high traffic high diesel, HTLD: high traffic low diesel, LTLD: low traffic low diesel.

**Table 2:** Estimated leakage area model parameters for older homes (built in 1979 or before).

House-Type	Parameter ^1^	Description	Estimate (95% CI)
Low-income	β_0_	Intercept	6.55 × 10^1^ (2.90 × 10^1^, 1.02 × 10^2^)
	β_1_	Year built	−3.40 × 10^−2^ (−5.29 × 10^−2^, −1.51 × 10^−2^)
	β_2_	Floor area	−7.33 × 10^−4^ (−9.34 × 10^−3^, 7.88 × 10^−3^)
Conventional	β_0_	Intercept	5.69 × 10^1^ (1.77 × 10^1^, 9.62 × 10^1^)
	β_1_	Year built	−2.91 × 10^−2^ (−4.91 × 10^−2^, −9.07 × 10^−3^)
	β_2_	Floor area	−5.65 × 10^−3^ (−1.39 × 10^−2^, 2.58 × 10^−3^)

Note: **^1^** β_0_ and β_1_ are dimensionless, β_2_ expressed in units of m^−2^.

**Table 3:** Literature-reported leakage area model parameters for newer homes (built after 1979).

House-Type	Parameter ^1^	Description	Value
Low-income	β_0_	Intercept	11.1
	β_1_	Year built	−5.37 × 10^−3^
	β_2_	Floor area	−4.18 × 10^−3^
Conventional	β_0_	Intercept	20.7
	β_1_	Year built	−1.07 × 10^−2^
	β_2_	Floor area	−2.20 × 10^−3^

Note: ^1^ β_0_ and β_1_ are dimensionless, β_2_ expressed in units of m^−2^.

**Figure 2:**
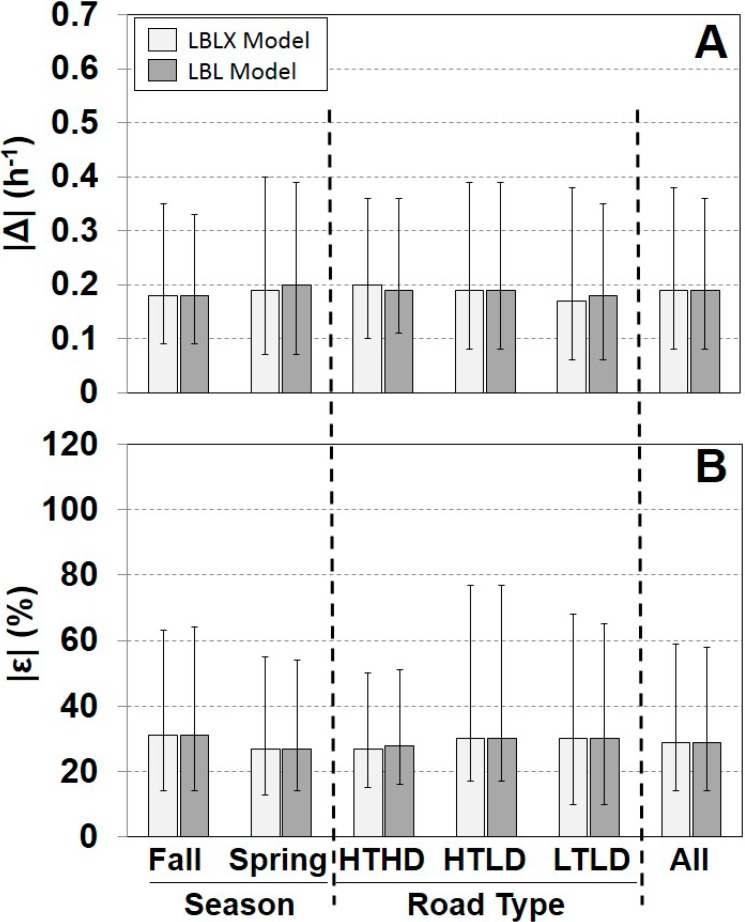
Comparison of absolute differences |Δ| (**A**) and relative differences |ε| (**B**) between individual modeled and measured AER for each model. Results are separated by season, road type, and across all days. Sample sizes are provided in Table 1. Shown are medians with 25th and 75th percentiles.

A comparison of the individual modeled and measured AERs is shown for each season, road type, and overall (Figure 2, Supplementary Material Figure S3). Overall, the LBLX and LBL showed similar results with the same median |ε| of 29%, and median |Δ| of 0.19 h^−1^. The LBLX and LBL models also showed similar |ε| quartiles for each season and road type. The LBLX model generally overestimated the AER with overall median ε of 6% and median Δ of 0.03 h^−1^ (Supplementary Material Figure S3). The LBL model also tends to overestimate the AER, but with a slightly smaller overall median ε of 5%. For the HTHD road type, the LBLX and LBL models underestimated the AER with overall median ε of −14% and −17%, respectively. For the two seasons and the HTLD and LTLD road types, the LBLX and LBL model tended to overestimate the AER.

**Figure 3:**
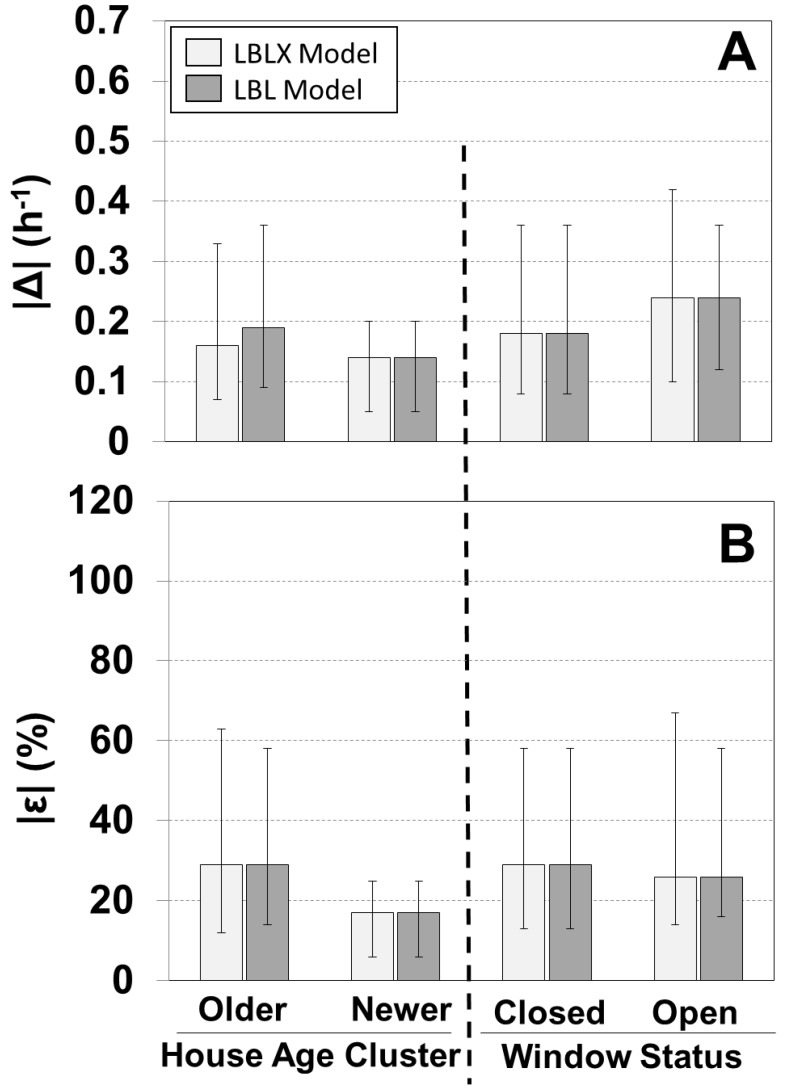
Comparison of absolute differences for |Δ| (**A**) and relative differences |ε| (**B**) between individual modeled and measured AER for the LBLX and LBL models. Results are separated by house age and window status. Sample sizes for 23 older homes and one newer home are 192 and 5, respectively, and for windows closed and open are 169 and 28, respectively. Shown are medians with 25th and 75th percentiles.

We evaluated the models for the older homes and the one newer home (Figure 3, Supplementary Material Figure S4). For the older homes, the LBLX and LBL models showed similar results with overall median |ε| of 29% and 29%, and median ε of 6% and 5%, respectively. Since windows were not opened in the newer home, the LBLX and LBL models had identical results with median |ε| of 17% and median ε of 6%.

A comparison of the individual modeled and measured AERs is shown for different window opening status (Figure 3, Supplementary Material Figure S4). The LBLX and LBL models are equivalent for days with windows closed, and therefore show identical results with median |ε| of 29% and median ε of 6%. For days with windows opened, the LBLX and LBL models showed similar results with identical overall median |ε| of 26%, and median |Δ| of 0.24 h^−1^. However, the LBLX model tends to bias the AER less than the LBL model with ε medians of 1% and −14%, respectively.

### 3.2. Model Predictions for NEXUS

**Figure 4:**
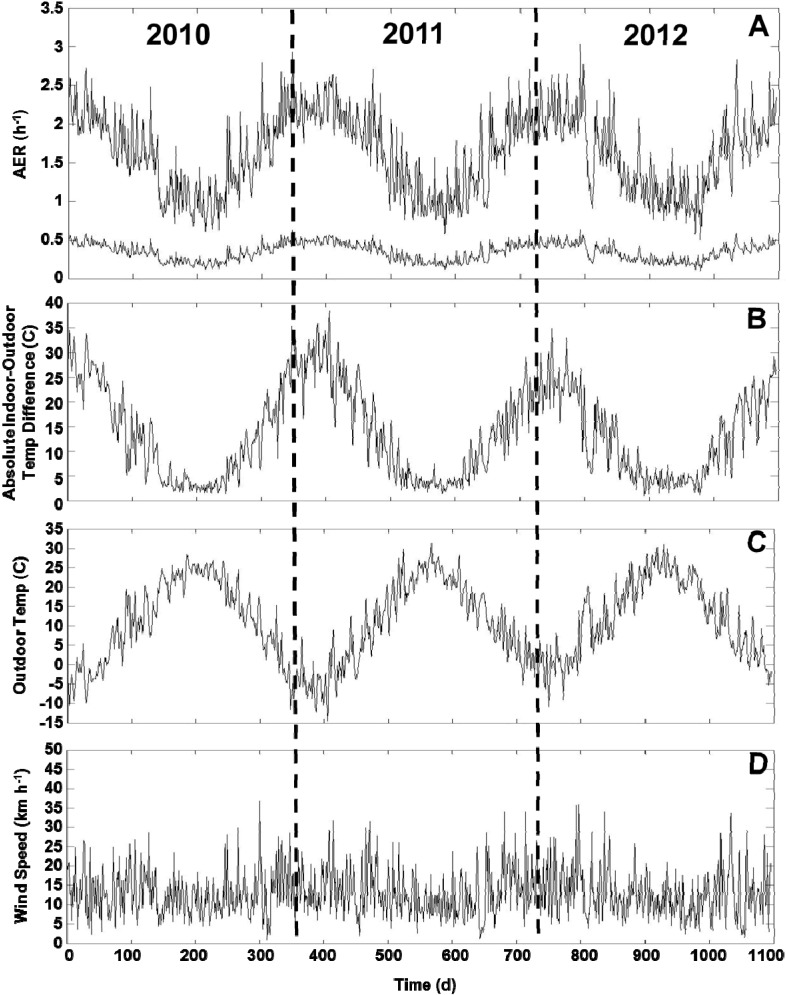
Time-course of AER predictions (**A**), absolute indoor-outdoor temperature differences (**B**), outdoor temperatures (**C**), and wind speeds (**D**) across the three years of health study. Two AER time-course plots correspond to homes with highest and lowest median AER predictions. Plots show daily 24 h average values across three years of health study from 1 January 2010 to 31 December 2012. AER oscillations correspond to indoor-outdoor temperature differences. AER transients of positive or negative spikes correspond primarily to wind speeds and secondarily to indoor-outdoor temperature differences.

**Figure 5:**
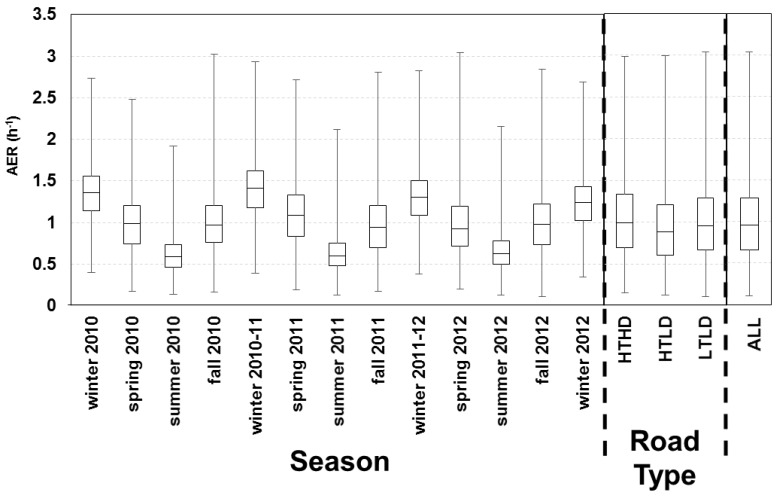
AER predictions for 213 homes across three years of health study with results for each season and road type. Boxes correspond to median, 25th and 75th percentiles; and whiskers correspond to minimum and maximum values. Winter includes December, January, and February; spring includes March, April, May; summer includes June, July, August; fall includes September, October, and November.

For applying the LBL model for the health study, we predicted the daily AER (24 h average) for all 213 homes across three years. Summary statistics are provided for the building characteristics (Supplementary Material Table S4). The variability of the daily indoor-outdoor temperature difference, outdoor temperature and wind speed is shown across three years (Figure 4B,D). The modeled AER varied between 0.11 h^−1^ (minimum) and 3.04 h^‑1^ (maximum) with 25th, 50th, and 75th percentiles of 0.66, 0.95, and 1.28 h^‑1^, respectively (Figure 5). The modeled AER time-course is shown for two homes: homes with highest and lowest median AER predictions (Figure 4A). The slow AER oscillations correspond to variations of the indoor-outdoor temperature differences (Figure 4). The brief AER transients (*i.e.*, positive and negative spikes) correspond primarily to the wind speed variations, and secondarily to indoor-outdoor temperature difference variations (Figure 4). The AER variability is shown for each season and road type (Figure 5). The median modeled AER was highest in the winters (1.36, 1.41, 1.31, and 1.24 h^‑1^ for the four consecutive winters) and lowest in the summers (0.59, 0.60, 0.63 h^‑1^ for the three consecutive summers). This seasonal variation corresponded to the median indoor-outdoor temperature differences highest in the winters (26.7, 27.8, 23.3, 22.2 ºC for the four consecutive winters) and lowest in the summers (0.8, 0.6, 0.0 ºC for the three consecutive summers), but did not correspond to the wind speeds, which did not vary between seasons The median wind speeds in winter (12.9, 14.5, 12.9, 12.9 km h^‑1^ for the four consecutive winters) and spring (12.9, 12.9, 12.9 km h^‑1^ for the three consecutive springs) were similar and often slightly higher than the wind speeds in the summer (11.3, 9.7, 11.3 km h^‑1^ for three consecutive summers) and fall (11.3, 12.9, 11.3 km h^‑1^ for the three consecutive falls). For the HTHD, HTLD, and LTLD road types, the modeled AER were similar with medians of 0.99, 0.89, and 0.96 h^‑1^, and interquartile ranges of 0.64, 0.60, and 0.62 h^‑1^, respectively.

**Figure 6:**
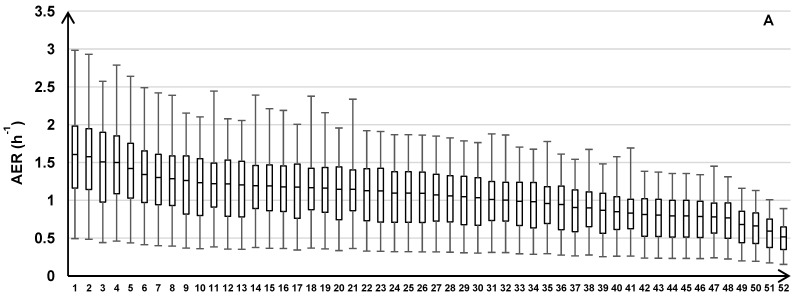

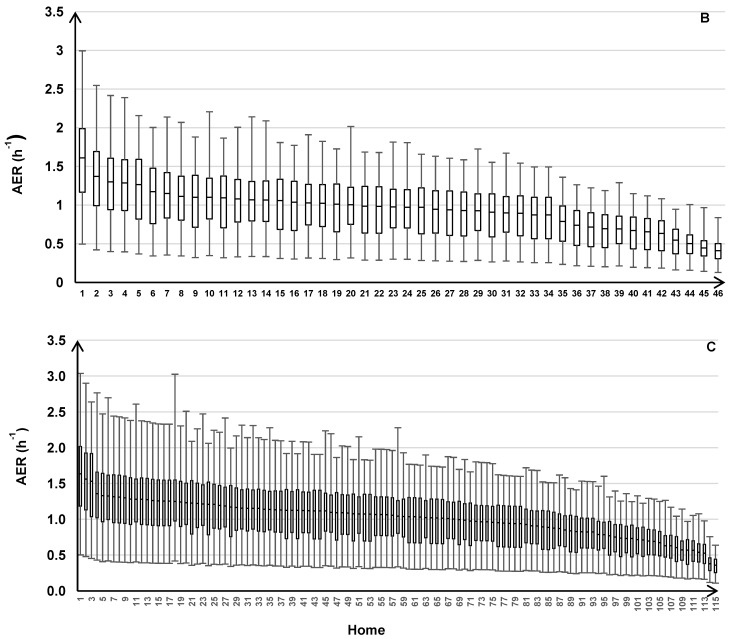
AER predictions for 213 homes across the three years of the health study with results for individual homes grouped by the three traffic categories: HTHD (**A**), HTLD (**B**), and LTLD (**C**). Box plots show median, 25th and 75th percentiles, and whiskers represent minimum and maximum values of 24 h average AER.

The variability of the AER predictions is shown for the individual homes within each road type (Figure 6). Across all road types, the modeled AER varied between 0.11 and 0.50 h^‑1^ for the minimums, 0.36 and 1.64 h^‑1^ for the medians, and 0.64 and 3.04 h^‑1^ for the maximums.

The temporal AER variability of individual homes tends to decrease with decreasing median AER (Figure 4A, Figure 6). To calculate the AER, the leakage area (constant across time) is multiplied by the stack and wind effects, which vary across time (Equation (4)). Therefore, homes with tighter building envelopes tend to have smaller model-predicted AER fluctuations, which are due to the temporal fluctuations of the stack and wind effects.

## 4. Discussion

Our goal was to develop daily AER predictions for each NEXUS participant home to provide improved exposure estimates for the health study. We used cross-validation to evaluate two models (LBL and LBLX), which predict residential AER from questionnaires and meteorology, with measured AERs from a subset of NEXUS homes. The daily modeled AER closely correspond to the measured AER with the same overall |ε| median of 29% for both the LBL and LBLX models. These results demonstrate that it is possible to apply these models for individual-level air pollution exposure assessments that require daily predictions of house-specific AER. However, the impact of applying these models for a health study in support of improving health effect estimates will depend not only on the accuracy of exposure predictions, but also on other factors such as the design of the health study [33,34].

We found considerable variation in measured AERs (range: 0.09–3.48 h^‑1^) and modeled AERs (range: 0.11–3.04 h^‑1^). Another study in central North Carolina showed similar variation in measured AERs (range: 0.09–3.17 h^‑1^) across 31 homes on seven consecutive days during the same two seasons (spring, fall) as the seasonal intensives in NEXUS [7]. This suggest that AER differences may be an important source of heterogeneity in the infiltration of outdoor air pollutants into homes and the resulting exposures, even for studies focused on within-city variations and for studies in different geographical locations. Using questionnaire and weather data, the LBLX and LBL models explained a substantial amount of the measured AER variation (R^2^ = 61% and 59%, respectively).

There is substantial temporal variation in the modeled AER that differs for each home based on the building envelope tightness. The home with the largest *A*_leak_ (*i.e.*, leakiest building envelope) had the highest median AER (1.64 h^‑1^) and largest AER range (0.50–3.04 h^‑1^) across time. The home with the smallest *A*_leak_ (*i.e.*, tightest building envelope) had the lowest median AER (0.36 h^‑1^) and smallest AER range (0.11–0.64 h^‑1^) across time.

This study demonstrates a novel health study design and modeling method designed to improve residential AER predictions for individual exposure assessments in health studies. This study is the first to use daily AER measurements and window opening data from a subset of homes for parameter estimation (*i.e.*, model calibration) and model evaluation, and then apply the calibrated model to predict the spatial and temporal variations of the AER for each participant’s home in a health study. This approach allowed us to estimate the uncertainty of the model parameters (e.g., based on the jackknife method) and the uncertainty of the model predictions (*i.e.*, based on the cross validation method), which can be important when the model is applied for health effect analyses [33].

We can compare our model performance using two alternative approaches for parameter estimation of *A*_leak_. First, we estimated parameters using both the 23 older homes and the one newer home combined instead of estimating parameters using only the 23 older homes, as described in the methods. Using this alternative method, the median |ε| for the one newer home increased from 17% to 91% (Supplementary Material Figure S5, Figure 3). Second, we used the literature-reported parameters for both the 23 older homes and one newer home instead of only for the newer home, as described in the methods. Using this alternative approach, the median |ε| for the older homes increased from 29% to 43%, the 25th percentile increased from 12% to 19%, and the 75th percentile increased from 63% to 131% (Supplementary Material Figure S5, Figure 3). This demonstrates the benefit of including AER measurements from a subset of homes, which represent the housing stock of homes in the same city as the health study, to reduce the AER model uncertainty.

We can compare the AER model evaluation with other studies. LBL model evaluations using whole-building pressurization measurements to determine the leakage area showed mean |ε| of 26%–46% [35] and 25% [36] for detached homes. For our implementation of the AER models, which uses a leakage area model, the LBL and LBLX models had mean |ε| of 43% and 48%, respectively for 31 detached homes across four seasons in central North Carolina [7]. In this study, the LBL and LBLX models both had a mean |ε| of 45%. Given the limitations of single-zone AER models (e.g., no internal resistance to airflow, no internal temperature or pressure differences) and the AER measurement error of the PFT method (accuracy of 20%–25%, precision of 5%–15% for occupied homes) [19,25,26], our LBL and LBLX model evaluations are reasonable, but their impact will depend on the particular application.

For parameter estimation, *T*_in_ was set to the 24 h average indoor temperature time-matched to the 24 h average AER measurements from a subset of homes. However, for predicting the daily AER for all homes across the three year study, *T*_in_ was set to a constant (24 ºC), which was the median indoor temperature measured in subset of homes. To investigate the impact of using a constant *T*_in_, we compared the LBL model predictions with *T*_in_ set to a lower and upper limit of 20 and 28 ºC, respectively. For *T*_in_ set to 20, 24, and 28 ºC, the minimum AER was 0.10, 0.11, and 0.16 h^−1^; the median AER was 0.88, 0.95, and 1.05 h^−1^; and the maximum AER was 3.00, 3.04, 3.12 h^−1^. Since these results are similar, we expect that setting *T*_in_ to 24 ºC does not have a substantial impact on the AER model predictions.

On days with open windows, similar model evaluation results were obtained for the LBLX model, which includes both leakage and natural ventilation, and the LBL model, which includes only leakage. Another study showed similar results for the LBLX and LBL models with AER measurements and window opening data from 31 homes in central North Carolina [7]. For 253 days with open windows across 4 consecutive seasons, the median |ε| was 41% and 48% for the LBLX and LBL models, respectively. For days with open windows, the LBL model slightly underestimates, the LBLX model slightly overestimates. Also, the LBL and LBLX models may perform similarly since windows may be opened more often on comfortable days with small indoor-outdoor temperature differences. Thus, the stack effect may be small on days with windows opened. Also, the stack effect can be reduced after windows are opened from a thermal equilibrium created between indoor and outdoor temperatures. These results suggest that our application of the LBL model, instead of the LBLX model, for the NEXUS health study is reasonable. In certain geographical locations (e.g., coastal regions) with high and persistent winds, comfortable outdoor temperatures across seasons, and frequent window opening; the LBLX model may provide substantially improved estimates as compared to the LBL model.

The temporal resolution of the AER is determined by the meteorological data. In this paper, we used hourly outdoor temperature and wind speed measurements to predict hourly AER, and then calculated 24 h averages to compare with the 24 h average AER measurements. To account for the diurnal variation of traffic-related air pollutants, we plan to use the hourly AER predictions combined with hourly residential outdoor concentration predictions to predict every NEXUS participant’s hourly residential indoor concentrations based on the dynamic mass balance model (Equation (3)) [4].

Since the AER is the key parameter for *F*_inf_ (Equation (2)), we can compare our AER models with a previously reported model used to predict *F*_inf_ of outdoor PM_2.5_ for individual homes in a health study [13]. The reported *F*_inf_ model is an empirical linear regression model that does not include the stack and wind effects, which are the driving forces for leakage and natural ventilation airflows. The *F*_inf_ model also does not account for differences in the leakage area between homes. In our study, we used the mechanistic LBL and LBLX models that include the stack and wind effects, and the building characteristics that modify the stack effect (*i.e.*, building height) and wind effect (*i.e.*, local wind sheltering and building height). Also, these AER models are linked to a building-specific leakage area model (Equation (5)). Furthermore, we estimated only a few parameters based on daily measurements, whereas the reported *F*_inf_ model required several parameters to be estimated based on two-week average measurements.

Most air pollution health studies use outdoor concentrations as an exposure surrogate. Under steady-state conditions, exposure *E* can be described by:

*E* = *C*_out_ss_(*f*_in_*F*_inf_ + (1 − *f*_in_)) (15)

where *f*_in_ is the fraction of time spent indoors. Therefore, *E* depends on the product of steady-state outdoor concentration *C*_out_ss_ and outdoor attenuation (*f*_in_*F*_inf_ + (1 − *f*_in_)). Since people spend more time indoors than outdoors (*i.e.*, *f*_in_ > (1 − *f*_in_)) [20], *F*_inf_ is a substantial component of outdoor attenuation. When *C*_out_ss_ is used as an exposure surrogate, the estimated health effect parameter is reduced (*i.e.*, biased towards the null) since it is the product of the toxicity (*i.e.*, true health effect) and outdoor attenuation [4]. Using *E* instead of *C*_out_ss_ in health studies should yield a less attenuated health effect estimate [37]. Since the LBL model inputs are relatively easy to obtain, our modeling approach can facilitate the estimation of *F*_inf_ to help support the use of *E* in health studies. Also, accounting for AER variability can reduce the uncertainty of *F*_inf_ and the resulting exposure in support of improving health effect estimates.

For exposure models, there are two components of measurement error [4,34]. The Berkson-like component of error results from using a model that has some sources of variation or exposure factors missing from the model. The classical‑like component of error is from uncertainty in the estimated model parameters. Both types of measurement error have an impact on health effect estimates. The Berkson error can increase confidence intervals of health effect estimates while classical error can lead to incorrect confidence intervals and biased health effect estimates [4,34]. Under a new measurement error correction method [33], Berkson-like error can also induce a bias. The method used in this study can minimize both types of errors. Our mechanistic AER models (*i.e.*, LBL and LBLX models) can reduce Berkson error, as compared to using empirical AER models that do not account for temporal variations due to the stack and wind effects [11]. Also, our model calibration with a subset of homes to improve the estimated parameters of the leakage area model can reduce classical error.

A limitation of this study is that mechanical ventilation could not be included in the AER predictions for the three year health study since it was not collected due to cost and participant burden considerations. We expect bathroom fans, outdoor-vented kitchen range hoods, and clothes dryers, which have low-intermediate airflows and are used intermittently, to have a small AER effect. Central heating and air conditioning (HVAC) systems in homes re-circulate indoor air with no outdoor air intake, but can have air duct leaks in unconditioned spaces (e.g., basements, attics) when operated [38]. However, none of the NEXUS homes had HVAC systems. Window/wall air conditioners also re-circulate indoor air, but can be operated with open outdoor vents. Other types of outdoor-vented fans include window fans and whole-house fans, which move outdoor air into the living space through open windows. Overall, we expect a large AER effect from window fans, whole-house fans, and window/wall air conditioners operated with open outdoor vents. Attic fans, which ventilate the attic space and not the living space with soffit or gable vents, are expected to have a small AER effect. The ability to quantify the impact of mechanical ventilation on the AER in this study is not possible since the variability of mechanical ventilation can be substantial due to various factors, which include the type of mechanical ventilation, frequency of use, and method of operation (e.g., open or closed outdoor vents for window/wall air conditioners).

Another limitation of this study is that the AER were measured in the spring and fall, with no measurements from the summer or winter due to cost. However, the leakage area model parameters, which were estimated from the AER measurements and applied for the older homes, are independent of the stack and wind effects that can vary seasonally. Therefore, we expect AER measurements from different seasons to have a small effect on the estimated parameters. In addition, a previous study that compared AER measurements with LBL and LBLX model predictions, which used the same literature-reported parameters that we applied for the newer homes in this study, showed similar results in all four seasons [7]. The LBL and LBLX models had median relative errors of 41% and 37% in spring, 45% and 44% in summer, 43% and 40% in fall, 39% and 39% in winter, respectively. Therefore, we expect the model performance in this study to be similar across the four seasons.

An additional limitation is the AER measurements used for parameter estimation were from a cluster of 23 older homes built between 1900 and 1969 (median 1942). Therefore, the estimated parameters were applied for the older homes in the health study, and literature-reported parameters were used for the newer homes in the health study. However, a previous study that compared AER measurements with LBL and LBLX model predictions [7], which used the same literature-reported parameters, showed results similar to those reported in this study, which used the estimated parameters. Based on 642 AER measurements from 31 homes built between 1922 and 2000 (median 1965), the median |ε| was 43% (0.17 h^−1^) and 40% (0.17 h^−1^) for the LBL and LBLX models, respectively [7]. In this study, the median |ε| was 29% (0.19 h^−1^) for both the LBL and LBLX models. Therefore, we expect the model performance in this study to be similar for the older and newer homes.

Another limitation is the small sample size (six homes) used to estimate parameters for conventional homes. This can increase uncertainty in the estimated parameters, and lead to more classical-like measurement error.

## 5. Conclusions

This study demonstrates the ability of using a novel method of integrating AER measurements and models to predict the large home-to-home (spatial) and temporal variability of residential AERs, which is an important determinant of exposure heterogeneity in air pollution health studies. Using AER measurements from a subset of homes, we calibrated, evaluated, and applied mechanistic AER models that agree closely to daily AER measurements and explain a substantial amount of the AER variation. Using this novel approach, NEXUS will be one of the first epidemiology studies to apply calibrated and home-specific AER models, and to include the spatial and temporal variations of AER for over 200 individual homes across multiple years into an exposure assessment. This capability will help to provide more accurate exposure estimates for epidemiological studies in support of improving risk estimates.

## Supplementary Files

Supplementary File 1
