# Supplementary material for: Modeling Spatial and Temporal Variability of Residential Air Exchange Rates for the Near-Road Exposures and Effects of Urban Air Pollutants Study (NEXUS)
Source: Int J Environ Res Public Health. 2014 Nov 7;11(11):11481–504. doi: 10.3390/ijerph111111481 (PMC4245625; doi:10.3390/ijerph111111481)
Supplement: Supplementary File 1 [file ijerph-11-11481-s001.pdf]

Modeling Spatial and Temporal Variability of Residential Air Exchange Rates for the Near-Road Exposures and Effects of Urban Air Pollutants Study (NEXUS)

Supplementary Materials

Method for calculation of jackknife estimate and confidence interval. Let θ be the parameter of interest and let $\hat{\theta}_1, \hat{\theta}_2, ..., \hat{\theta}_n$ be the estimates of θ based on n subsamples, each of size $n-1$. The jackknife estimate of θ is the arithmetic average given by:

$$\hat{\theta}_J = \frac{1}{n}\sum_{i=1}^{n}\hat{\theta}_i \quad \text{(S1)}$$

The $100(1-\alpha)$ percent confidence interval (CI) of the jackknife estimate is:

$$\text{CI} = \hat{\theta}_J \pm t_{\alpha/2,n-1}\,\hat{\sigma}_J \quad \text{(S2)}$$

where $\hat{\sigma}_J$ is the standard error defined as:

$$\hat{\sigma}_J = \left[\frac{n-1}{n}\sum_{i=1}^{n}\left(\hat{\theta}_i - \hat{\theta}_J\right)^2\right]^{0.5} \quad \text{(S3)}$$

where $t_{\alpha/2,n-1}$ is the upper $\alpha/2$ percentage point of the t-distribution with n-1 degrees of freedom. For the 95 percent confidence interval with n = 17 (low-income homes), $t_{0.025,16}$ = 2.120, and with n = 6 (conventional homes), $t_{0.025,5}$ = 2.571.

Figure S1. Year built for 24 homes with measured AER (**A**) and all 213 homes (**B**).

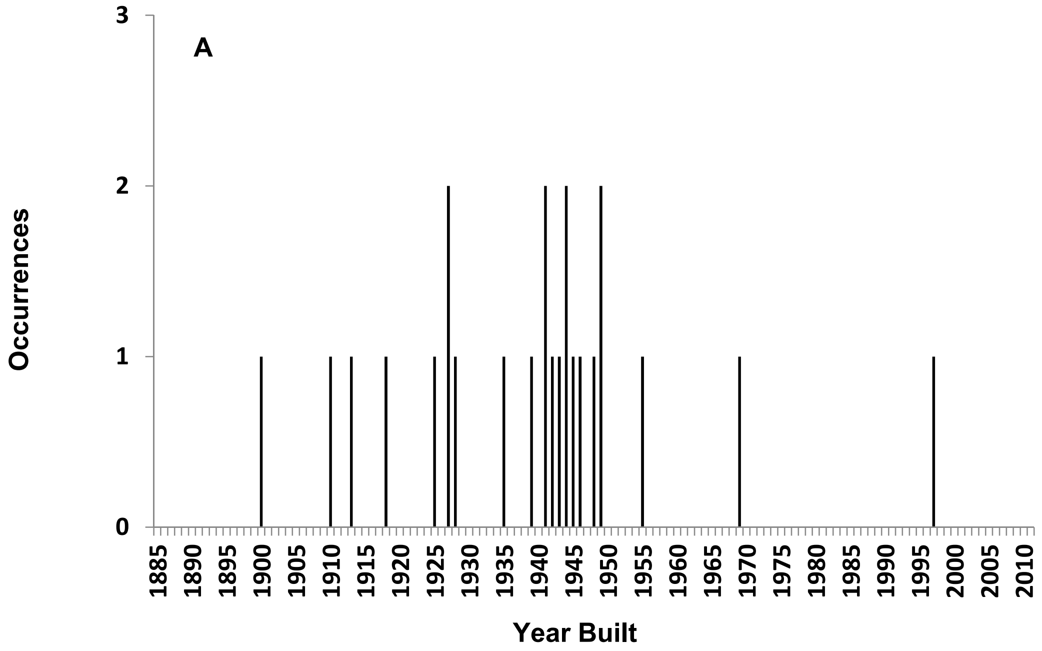

Figure S1. *Cont.*

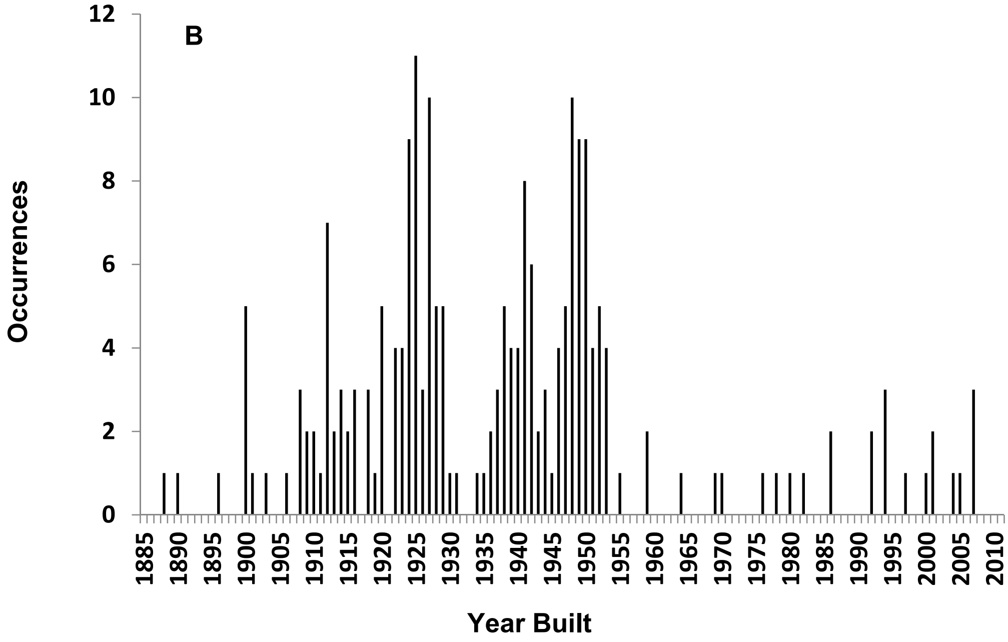

Figure S2. Scatter plots of LBLX and LBL model-predicted and measured AER for each home. The points are individual AER values for each home. Points above and below the 1:1 line indicate model overestimation and underestimation, respectively.

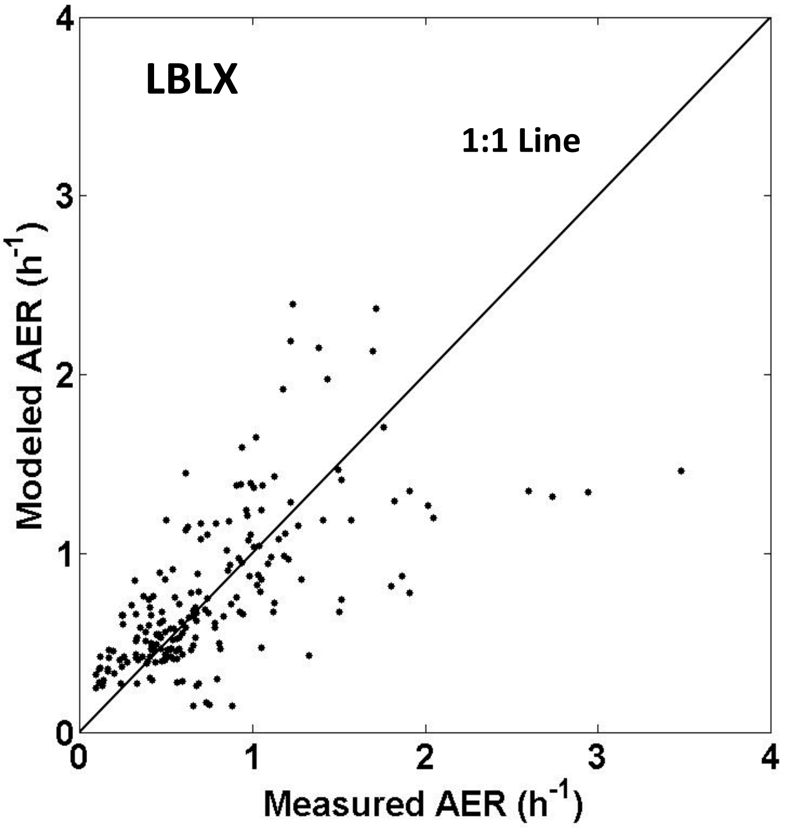

Figure S2. *Cont.*

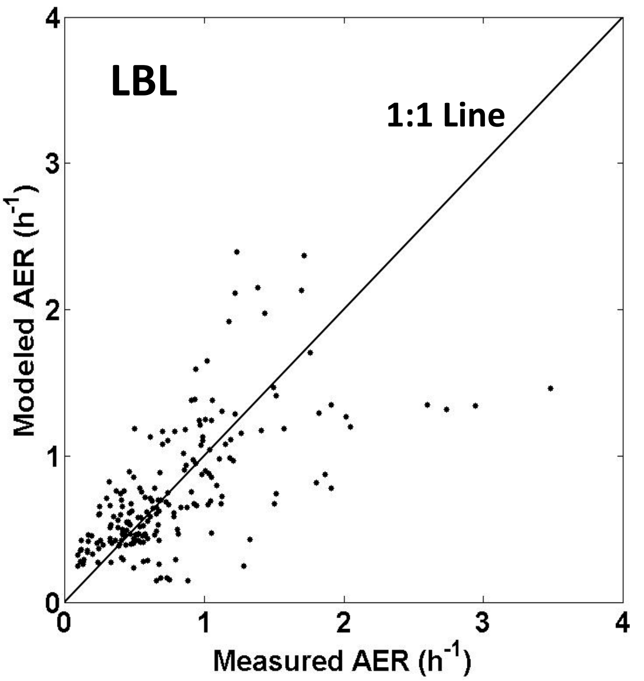

Figure S3. Comparison of signed differences for Δ (**A**) and ε (**B**) between individual modeled and measured AER for each model. Results are separated by season, road type, and across all days. Shown are medians with 25th and 75th percentiles.

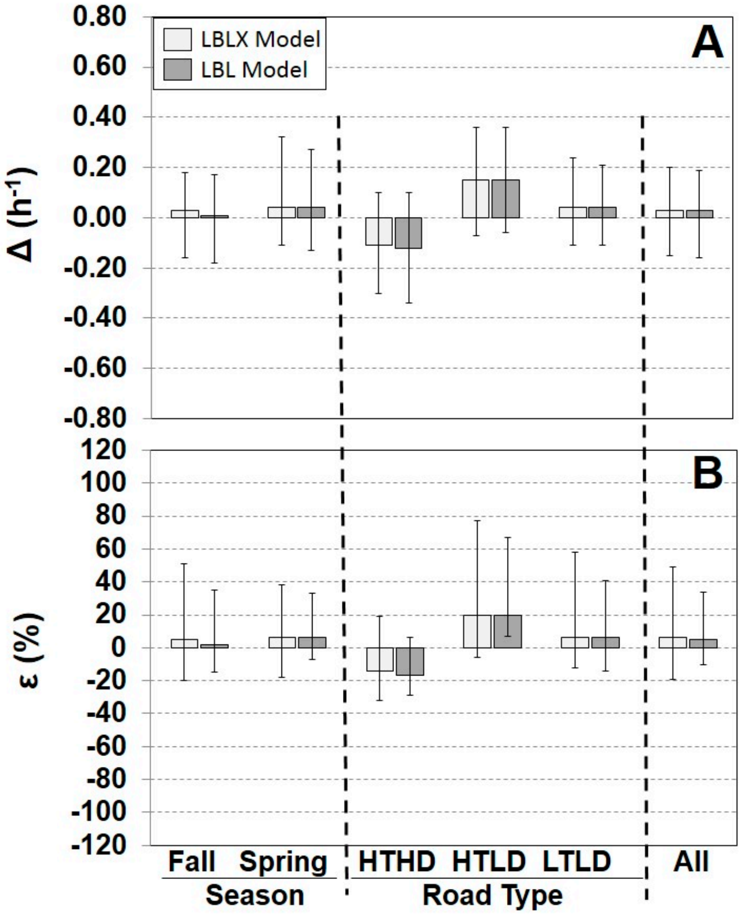

Figure S4. Comparison of signed differences for Δ (**A**) and ε (**B**) between individual modeled and measured AER for the LBLX and LBL models. Results are separated by house age and window status. Shown are medians with 25th and 75th percentiles.

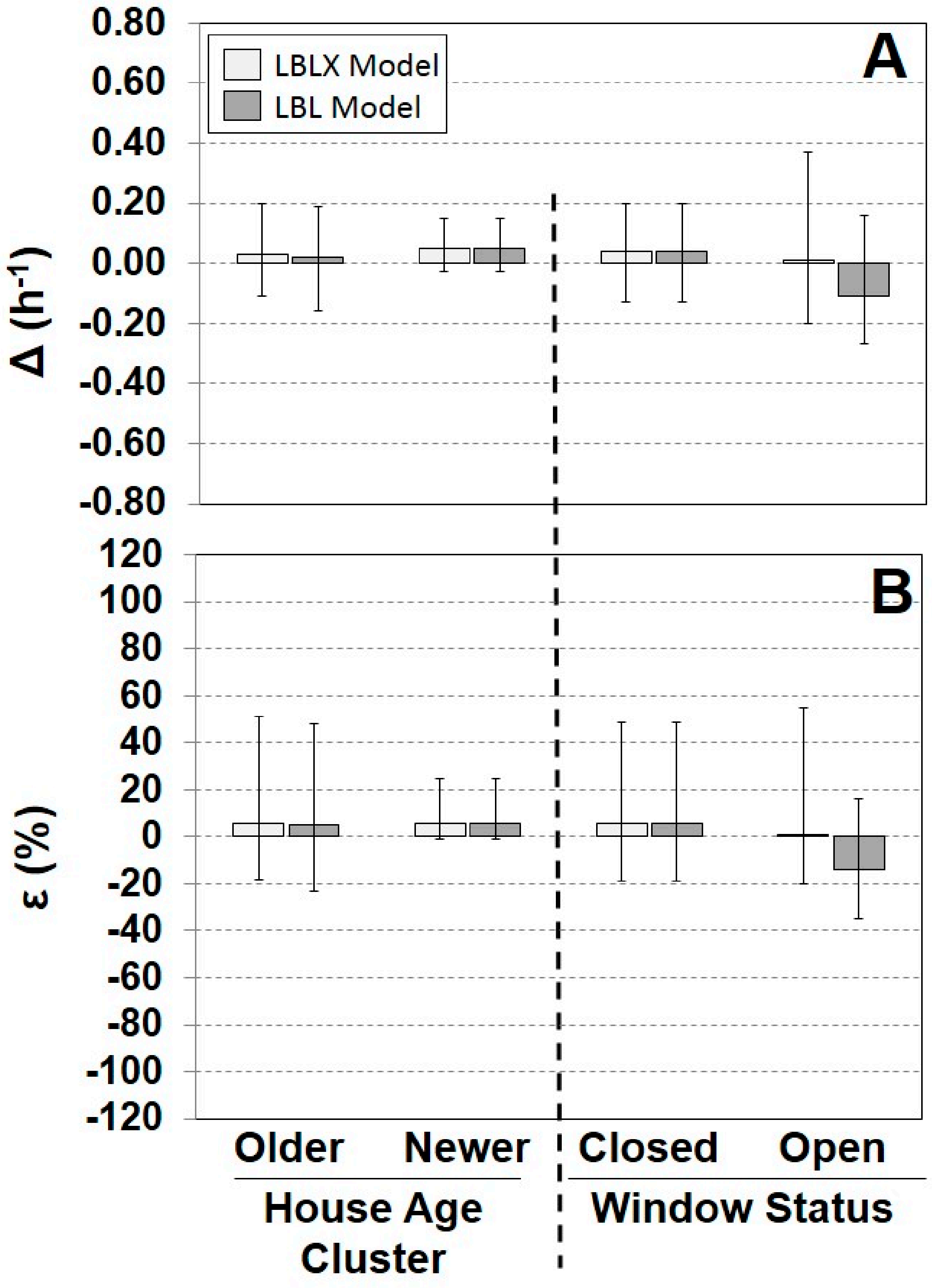

Figure S5. Comparison of absolute differences for |ε| between individual modeled and measured AER for the LBLX model. Results are shown for parameters estimated using one house age cluster for the leakage area model (estimated parameters), and results from literature-reported paramters (fixed parameters). Results are separated by house age. Shown are medians with 25th and 75th percentiles.

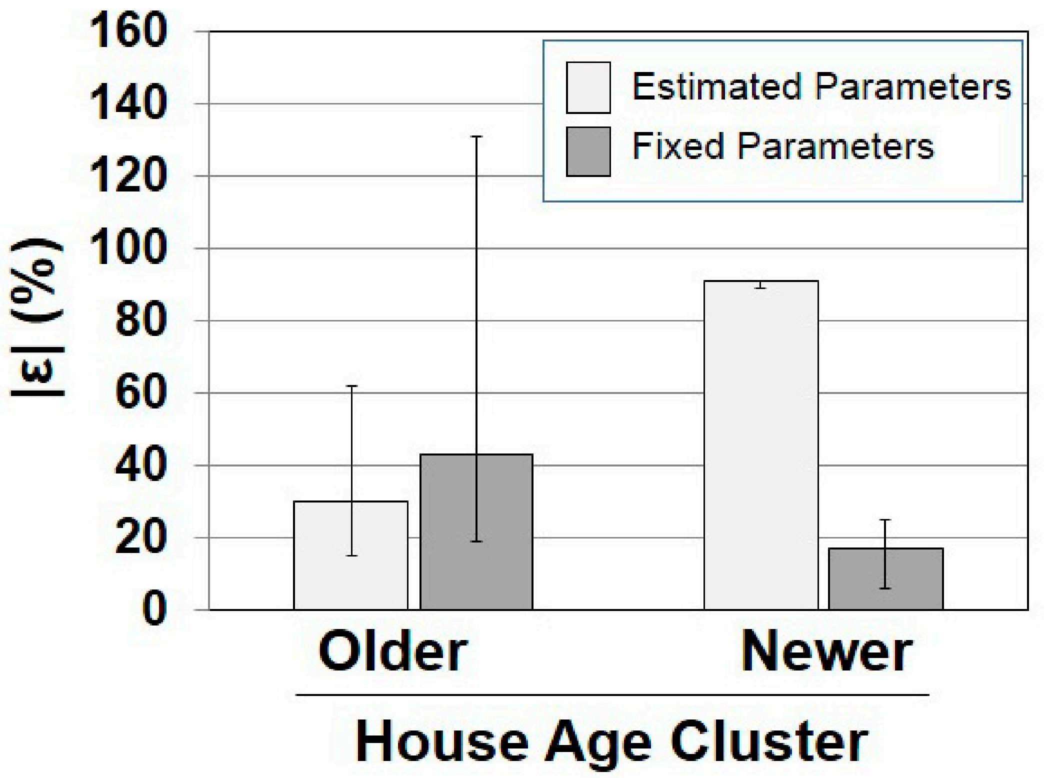

Table S1. Stack coefficient k_s ((L/s)^2/(cm^4 × K)).

Coefficient [1]	House Height (Stories)		
	**One**	**Two**	**Three**
Stack coefficient	0.000145	0.000290	0.000435

Note: [1] ASHRAE Handbook-Fundamentals, 2009 [1].

Table S2. Wind coefficient kw ((L/s)^2/(cm^4 × (m/s)^2))).

Shelter Class [1]	House Height (Stories)		
	**One**	**Two**	**Three**
1	0.000319	0.000420	0.000494
2	0.000246	0.000325	0.000382
3	0.000174	0.000231	0.000271
4	0.000104	0.000137	0.000161
5	0.000032	0.000042	0.000049

Note: [1] ASHRAE Handbook-Fundamentals, 2009 [1].

Table S3. Local sheltering.

Shelter Class [1]	Description [1]
1	No obstructions or local sheltering
2	Typical shelter for an isolated rural house
3	Typical shelter caused by other buildings across street from building under study
4	Typical shelter for urban buildings on larger lots where sheltering obstacles are more than one building height away
5	Typical shelter produced by buildings or other structures immediately adjacent (closer than one building height): e.g., neighboring houses on same side of street, trees, bushes, *etc*.

Note: [1] ASHRAE Handbook-Fundamentals, 2009 [1].

Table S4. Summary statistics of building characteristics for 24 homes used for model evaluation and 213 homes used for model prediction.

Model Input	Evaluation or Prediction	Number of Homes	Value (Year Built, Floor Area)						
			Mean	SD	Min	p25	p50	p75	Max
Year built	Evaluation	24	1939	20	1900	1927	1942	1948	1997
	Prediction	213	1938	24	1888	1924	1938	1949	2007
Floor area (m^2)	Evaluation	24	139	40	63	115	133	175	230
	Prediction	213	117	44	36	81	112	139	307
Housing-type									
Low-income	Evaluation	18							
	Prediction	185							
Conventional	Evaluation	6							
	Prediction	28							
Number of stories									
One-story	Evaluation	2							
	Prediction	30							
Two-stories	Evaluation	21							
	Prediction	178							
Three-stories	Evaluation	1							
	Prediction	5							
Local Sheltering									
Class 2	Evaluation	1							
	Prediction	1							
Class 3	Evaluation	3							
	Prediction	13							
Class 4	Evaluation	4							
	Prediction	106							
Class 5	Evaluation	16							
	Prediction	93							

Table S5. Summary statistics of LBLX modeled air exchange rates (24 h average) for 24 homes with air exchange rate measurements.

**Season: Year [1] Road Type, Window Status**	**Number of Homes**	**Number of Days Windows Opened [2]**	**Air Exchange Rates (h^{-1})**											
			**Sample Size**	**Mean**	**SD**	**Min**	**p5**	**p10**	**p25**	**p50**	**p75**	**p90**	**p95**	**Max**
Fall 2010	24	19 (16%)	119	0.72	0.43	0.15	0.26	0.28	0.42	0.60	0.97	1.35	1.41	2.39
Spring 2011	17	9 (12%)	78	0.85	0.45	0.39	0.40	0.42	0.46	0.72	1.11	1.44	1.83	2.37
HTHD [3]	7	12 (22%)	55	0.78	0.38	0.26	0.28	0.29	0.44	0.74	1.14	1.35	1.39	1.46
HTLD [3]	5	2 (5%)	44	0.80	0.46	0.25	0.27	0.29	0.39	0.69	1.17	1.39	1.61	1.92
LTLD [3]	12	14 (14%)	98	0.75	0.47	0.15	0.29	0.40	0.46	0.65	0.87	1.35	2.07	2.39
Windows closed	14	0	169	0.74	0.44	0.15	0.27	0.32	0.43	0.62	0.98	1.34	1.47	2.39
Windows open	10	28 (100%)	28	0.91	0.44	0.27	0.29	0.37	0.62	0.83	1.17	1.45	1.75	2.19
All	24	28 (14%)	197	0.77	0.44	0.15	0.27	0.33	0.45	0.65	0.99	1.36	1.55	2.39

Notes: [1] Fall: September, October, and November; spring: March, April, and May; [2] Percentage of days windows opened relative to corresponding sample size are shown in parentheses; [3] HTHD: high traffic high diesel, HTLD: high traffic low diesel, LTLD: low traffic low diesel.

Table S6. Summary statistics of LBL modeled air exchange rates (24 h average) for 24 homes with air exchange rate measurements.

**Season: Year [1] Road Type, Window Status**	**Number of Homes**	**Number of Days Windows Opened [2]**	**Air Exchange Rates (h^{-1})**											
			**Sample Size**	**Mean**	**SD**	**Min**	**p5**	**p10**	**p25**	**p50**	**p75**	**p90**	**p95**	**Max**
Fall 2010	24	19 (16%)	119	0.69	0.42	0.15	0.24	0.27	0.40	0.59	0.90	1.30	1.38	2.39
Spring 2011	17	9 (12%)	78	0.83	0.45	0.39	0.40	0.42	0.46	0.71	1.08	1.40	1.83	2.37
HTHD [3]	7	12 (22%)	55	0.74	0.38	0.24	0.26	0.28	0.42	0.67	1.12	1.32	1.35	1.46
HTLD [3]	5	2 (5%)	44	0.80	0.46	0.25	0.27	0.29	0.39	0.69	1.17	1.39	1.61	1.92
LTLD [3]	12	14 (14%)	98	0.72	0.46	0.15	0.23	0.40	0.46	0.62	0.78	1.25	2.06	2.39
Windows closed	14	0	169	0.74	0.44	0.15	0.27	0.32	0.43	0.62	0.98	1.34	1.47	2.39
Windows open	10	28 (100%)	28	0.76	0.44	0.17	0.23	0.26	0.45	0.69	0.88	1.29	1.75	2.11
All	24	28 (14%)	197	0.75	0.44	0.15	0.26	0.31	0.43	0.64	0.97	1.32	1.55	2.39

Notes: [1] Fall: September, October, and November; spring: March, April, and May; [2] Percentage of days windows opened relative to corresponding sample size are shown in parentheses; [3] HTHD: high traffic high diesel, HTLD: high traffic low diesel, LTLD: low traffic low diesel.

Reference

1. *The 2009 ASHRAE Handbook-Fundamentals*; American Society of Heating, Refrigerating, and Air Conditioning Engineers (ASHRAE): Atlanta, GA, USA, 2009.

© 2014 by the authors; licensee MDPI, Basel, Switzerland. This article is an open access article distributed under the terms and conditions of the Creative Commons Attribution license (http://creativecommons.org/licenses/by/4.0/).
